# Fishing for Prion Protein Function

**DOI:** 10.1371/journal.pbio.1000075

**Published:** 2009-03-31

**Authors:** Roberto Chiesa, David A Harris

## Abstract

The prion protein is infamous for its role in devastating neurological diseases, but its normal, physiological function has remained mysterious. A new study uses the experimentally tractable zebrafish model to obtain fresh clues to this puzzle.

The prion protein (PrP) is a membrane-anchored, neuronal glycoprotein whose normal function is uncertain, but which plays a crucial role in prion diseases, a class of fatal neurodegenerative disorders of humans and animals [1]. Bovine spongiform encephalopathy (“mad cow disease”) and kuru, which are transmitted by eating contaminated tissues, are the best known examples of these disorders, which also occur in inherited and sporadic forms. In prion diseases, the normal, endogenous form of PrP (PrP^C^) undergoes transformation to a conformationally altered version (PrP^Sc^) that accumulates in the brain as sticky, insoluble aggregates. This process leads to neuronal dysfunction and progressive neurodegeneration, for which there is no effective treatment. Unlike other neurodegenerative disorders like Alzheimer's disease that are also due to protein misfolding, prion diseases are unique because they are transmissible. Prion propagation occurs by an unusual “protein-only” mechanism in which PrP^Sc^ imprints its aberrant conformation onto endogenous PrP^C^ molecules. Similar protein-based transmission of biological information has been described in other organisms such as yeast and fungi [2], and may well turn out to be widespread in nature.

In the last two decades, an impressive number of studies has investigated nearly every aspect of the prion phenomenon. Nevertheless, we still have very little understanding of how PrP misfolding causes neuronal dysfunction and death [3]. PrP knockout mice, in which the gene encoding PrP has been deleted, do not develop symptoms of prion disease, suggesting that pathogenesis may not be due simply to loss of an essential function of PrP^C^ upon its conversion to PrP^Sc^ [4–6]. Rather, it is commonly assumed that prion diseases result from a novel toxic activity acquired by PrP^Sc^, analogous to the mechanism proposed for other dominantly inherited neurodegenerative disorders. Interestingly, neurons lacking endogenous, membrane-anchored PrP^C^ seem to be resistant to the pathogenic effects of extracellular PrP^Sc^ [7–9]. This result suggests a connection between the neurotoxicity of PrP^Sc^ and the normal function of PrP^C^ on the cell surface [10]. For example, when PrP^C^ misfolds, its physiological activity might be altered as a consequence of oligomerization or abnormal interactions with partner proteins, resulting in generation of a toxic signal that is transmitted to the cell interior (Figure 1). Thus, ascertaining the normal function of PrP^C^ and identifying its cellular interactors will be essential for understanding the molecular pathogenesis of prion diseases.

**Figure 1:**
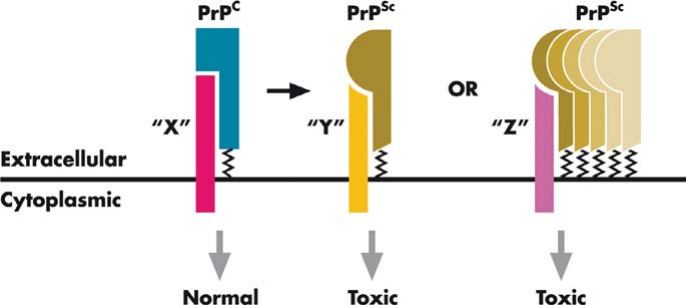
A Role for PrP^C^ Function in Prion Diseases PrP^C^ on the cell surface performs its normal function by associating with a hypothetical transmembrane interactor (“X”). In the disease state, PrP^Sc^ (or a misfolded intermediate) initiates a toxic signal by associating with different interactors (“Y” or “Z”), possibly as a result of oligomerization.

## Approaches and Challenges To Unraveling PrP Function

Genetic ablation of PrP expression in mice, either prenatally or postnatally, produces relatively little phenotypic effect, other than an inability to propagate prions [11]. Although PrP knockout mice display no major anatomical or developmental defects, a bewildering variety of subtle abnormalities have been described in these mice (reviewed in [12]). These include altered circadian rhythms [13] and olfaction [14], abnormalities in neuronal transmission and electrical activity [15], defective proliferation and differentiation of neural precursor cells [16] and hematopoietic stem cells [17], increased sensitivity to hypoxia, ischemia, and seizures [18], and enhanced resistance to microbial infections [19]. Although intriguing, none of these reported abnormalities has provided a definitive clue to the normal function of PrP^C^.

Studies on the cell biology of PrP^C^ have also failed to provide an unequivocal lead. Similar to other membrane glycoproteins, PrP^C^ is synthesized in the rough endoplasmic reticulum, transits the Golgi, and is delivered to the cell surface, where it resides in lipid rafts [20]. Some PrP^C^ molecules are then transferred to clathrin-coated pits, from which they undergo endocytosis and recycling. The cellular localization of PrP^C^ would be consistent with a number of different functions, including roles as a membrane receptor, adhesion molecule, or transporter. Perhaps the physiological activity that has emerged most frequently from a number of different cell culture studies is the ability of PrP^C^ to provide protection against various kinds of cellular stress, including oxidative damage, that normally induce cell death [21]. This activity would be consistent with some of the phenotypes observed in PrP knockout mice, such as increased sensitivity to hypoxia and ischemia.

A powerful strategy for elucidating the physiological function of PrP^C^ would be to identify other cellular proteins with which PrP^C^ interacts. Several candidate binding partners of PrP have been identified using yeast two-hybrid or biochemical approaches, including low-density lipoprotein receptor-related protein 1, neural cell adhesion molecule, stress-inducible protein 1, laminin receptor precursor, Bcl-2, and the potassium channel TREK-1 (reviewed in [21]). In most cases, however, the physiological relevance of the proposed interactions remains undocumented.

Recent studies demonstrate that selectively deleting certain sequence domains of PrP unleashes a powerful neurotoxic signal, possibly as a result of alterations in the physiological activity normally performed by PrP^C^. Transgenic mice expressing PrP molecules that lack portions of the unstructured N-terminus or central hydrophobic region display a dramatic neurodegenerative phenotype characterized by cerebellar degeneration and early death [22–24]. Amazingly, this phenotype is almost completely reverted by co-expression of full-length PrP, suggesting that the wild-type and deleted molecules interact in an antagonistic manner, either by complexing with each other or by competing for binding to a hypothetical membrane receptor. Working out the molecular details of these interactions will likely provide important insights into how PrP^C^ controls neuronal death and survival.

In principle, the use of non-vertebrate model organisms that can be genetically manipulated would greatly facilitate functional analysis of PrP. However, the systems normally used, including the yeast Saccharomyces cerevisiae, the nematode Caenorhabditis elegans, or the fruit fly Drosophila melanogaster, lack PrP homologous genes, precluding the possibility of carrying out a loss-of-function analysis. Attempts to model PrP-related phenotypes in these organisms by introducing heterologous PrP transgenes have generally been unrewarding, possibly because the required PrP-interacting proteins are not present.

Recently, PrP-related genes have been identified in zebrafish, thereby bringing this model organism to the attention of prion researchers. The paper by Edward Málaga-Trillo et al. in this issue of *PLoS Biology* [25] provides the first evidence for a strong PrP loss-of-function phenotype in a vertebrate system, offering potentially important new information on the physiological role of PrP.

## A Glue Function for PrP

The tiny zebrafish Danio rerio has become a powerful tool for studying vertebrate development. Among the many advantages of this species is that it can be easily observed and manipulated experimentally. Zebrafish eggs are clear, and develop outside of the mother's body, allowing scientists to watch them grow into a newly formed fish under a microscope. In addition, zebrafish are attractive from a genetic standpoint. Screens for mutant phenotypes are readily performed, and gene expression can be effectively knocked down, or exogenous genes expressed, by microinjecting the early embryo with antisense oligonucleotides or synthetic mRNAs, respectively. In addition, genetically modified cells can be transplanted into host embryos to analyze their behavior at different developmental stages, or to ask how mutant cells behave in wild-type embryos. Zebrafish have already been used successfully to model several human pathologies [26].

Because zebrafish are more closely related to humans than yeasts, nematodes, or fruit flies, they may be more useful for studying the function of a recently evolved protein like PrP. Like humans, zebrafish has its own PrP. In fact, two PrP-related genes, *PrP-1* and *PrP-2*, have been identified that encode proteins sharing key structural features with mammalian PrP, including a signal peptide, a series of repeat sequences in the N-terminal region, a central hydrophobic domain, and a disulfide bond in the C-terminus [27,28]. The globular C-terminal domain is predicted to have the same arrangement of a-helices and ß-strands as mammalian PrP. Moreover, zebrafish PrPs are glycosylated, and they contain a GPI anchor that attaches them to the cell surface [29].

What is the function of zebrafish PrP? To answer this question, Málaga-Trillo and colleagues began by studying the expression profile of the two genes, and found that *PrP-1* and *-2* have complementary expression patterns during fish development. While *PrP-1* transcripts are expressed ubiquitously and at high level in the early stages of embryogenesis, *PrP-2* is up-regulated later in the developing nervous system, suggesting that the two genes may fulfill different roles in zebrafish life.

To test this prediction, the authors knocked down *PrP-1* or *-2* expression by microinjecting morpholino antisense oligonucleotides into embryos at the 1–4 cell stage. These embryos (morphants) exhibited a striking morphological phenotype. PrP-1 knockdown embryos failed to carry out gastrulation, revealing an essential role for PrP-1 in the early phase of the fish development. In contrast, PrP-2 knockdown embryos underwent normal gastrulation and survived until the early larval stage. However, the larvae displayed morphological defects in the head, specifically malformed brains and eyes, consistent with a role of the *PrP-2* gene in neural differentiation and brain morphogenesis. The PrP-1 morphant phenotype (gastrulation arrest) could be fully rescued by microinjection of PrP-1 mRNA, and was partially suppressed by PrP-2 and mouse PrP mRNAs, suggesting functional conservation among all three PrP proteins.

But what is the functional activity of PrP whose absence produces such striking developmental abnormalities? To try and answer this question, the authors first investigated the cellular distribution of fish PrPs. By analyzing the localization of a series of PrP-EGFP (enhanced green fluorescent protein) chimeras in transfected mammalian cells and zebrafish embryos, they found that both PrP-1 and PrP-2 accumulated on the cell surface in regions of cell contact, with PrP-1 being restricted almost exclusively to these areas. Interestingly, mouse PrP was also concentrated at contact points. These observations led the authors to hypothesize that PrP-1 could play a role in cell–cell communication, a function that might be shared by mammalian PrP forms. Morphological examination of the PrP-1 morphant showed, in fact, that the developmental arrest was preceded by a marked decrease in tissue integrity, due to loss of embryonic cell adhesion.

A role for PrP-1 in cell adhesion was confirmed by the inability of embryonic cells lacking PrP-1 to form aggregates in culture. Moreover, embryonic cells from PrP-1 morphants could not establish normal cell contacts when grafted into wild-type embryos, indicating that the adhesion defect was cell autonomous, and could not be corrected by the normal cellular environment of the host embryos.

What is the molecular mechanism underlying the adhesion defects observed in these experiments? During gastrulation, cell adhesion is dynamically maintained by homophilic interactions of cadherins. Cadherins are a group of type-1 transmembrane proteins that play important roles in cell adhesion, ensuring that cells within tissues are bound together [30]. They are dependent on calcium ions to function; hence their name. While the cadherin extracellular domain is responsible for cell–cell interactions, the intracellular domain binds to the actin cytoskeleton via molecules known as catenins.

The authors found striking abnormalities of cadherin distribution in PrP-1 morphant embryos. E-cadherin (the cadherin isoform expressed in the early embryo) and ß-catenin showed an abnormal intracellular distribution, and the actin cytoskeleton was disorganized. Biochemical investigation showed a reduction in the amount of the mature E-cadherin isoform (the one that is exposed on the cell surface), and an increase in the amount of the intracellular, immature form. In addition, the amount of E-cadherin colocalizing with a particular class of intracellular vesicles was significantly increased in PrP-1 morphants, indicating reduced E-cadherin trafficking to the plasma membrane. Thus, PrP-1 may modulate the function of E-cadherin by regulating its processing and/or transport to the cell surface.

The authors also showed that local accumulation of E-cadherin and ß-catenin at newly formed cell contacts required PrP-1, and that this process was accompanied by accumulation of Fyn tyrosine kinase and tyrosine-phosphorylated proteins at the contact points. These observations suggest that regulation of E-cadherin localization by PrP-1 may involve a signal transduction mechanism, including activation of Src-family tyrosine kinases.

In addition to demonstrating a role for PrP in regulating cadherin-mediated adhesion, Málaga-Trillo et al. also show that PrP has its own, intrinsic adhesive properties. Drosophila Schneider 2 (S2) cells are normally non-adhesive, and grow in suspension as individual cells. However, if manipulated to express cell adhesion molecules, S2 cells can form multicellular aggregates. Since S2 cells do not express their own PrP, Málaga-Trillo et al. could directly test the effect of exogenous PrP expression on cell adhesion. This experiment showed that PrP expression promotes aggregation of S2 cells, and that PrP accumulates at adhesive sites. Similar results were obtained when S2 cells were transfected with PrP from other species (frog, chicken, or mouse), suggesting that the propensity for homophilic interactions is a general property of PrP, and that cross-species interactions are also possible. In additional experiments, the authors showed that PrP-mediated adhesion was associated with tyrosine kinase-based signal transduction events occurring within specialized domains of the plasma membrane.

**Figure 2:**
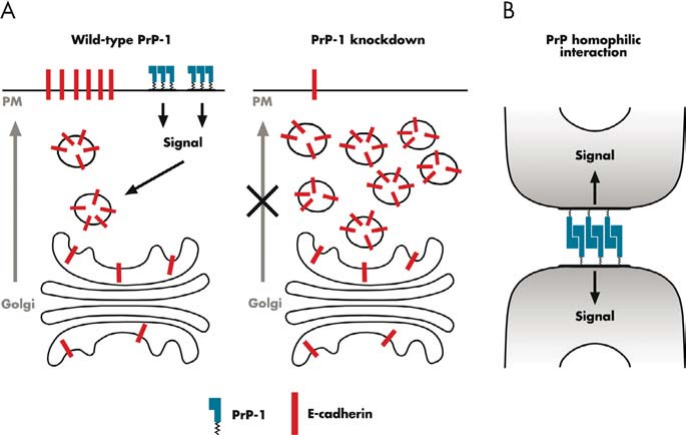
Two Roles for PrP in Cell Adhesion (A) In wild-type zebrafish (left), PrP-1 promotes proper delivery of E-cadherin from the Golgi to the plasma membrane (PM), possibly via activation of a signal transduction cascade involving Src-family tyrosine kinases. In morphant fish lacking PrP-1 (right), E-cadherin accumulates in intracellular vesicles, resulting in reduced delivery to the plasma membrane. As a result, Ca^+2^-dependent, cadherin-mediated cell adhesion is impaired. (B) PrP molecules on adjacent cells undergo homophilic interactions that promote cell adhesion in a Ca^+2^-independent manner, at the same time generating an intracellular signal involving tyrosine phosphorylation. The PrP functions depicted in the two panels of this figure could be linked, if the intracellular signal generated by homophilic binding of PrP molecules (B) regulates cadherin trafficking (A).

In summary, the study by Málaga-Trillo et al. indicates a role for zebrafish PrP-1 in modulating calcium-dependent cell adhesion in the developing fish embryo, through regulation of E-cadherin processing and/or trafficking (Figure 2A). In addition, their work shows that PrP-1 itself, and possibly mammalian PrPs, can act as calcium-independent, homophilic adhesion molecules (Figure 2B).

## Implications

The paper by Málaga-Trillo et al. is important for several reasons. First, it provides the first example of a dramatic phenotype caused by the absence of PrP. This is clearly different from the mild deficits described in PrP knockout mice. Second, the loss-of-function phenotype can be partially rescued by PrP from other species, including mammals, highlighting an evolutionarily conserved function for the protein. Third, the authors have shown that the knockdown phenotype is associated with a specific cellular deficit (abnormalities in cell adhesion), and in addition they have provided evidence that a transmembrane signaling function for PrP may play a role in the adhesion-promoting activities of the protein. Finally, this work is also important because it uses a simple animal model, which is amenable to genetic manipulation.

Clearly, much remains to be done to pursue these interesting observations. This study focuses primarily on the function of PrP-1, but it will be important now to analyze the role of PrP-2, which, based on its expression profile and morphant phenotype, is probably more closely related to mammalian PrP in terms of its physiological function in the brain. While there is evidence from this study that PrP-2 can partially substitute for PrP-1 in rescuing the morphant phenotype, it is possible that PrP-1 normally fulfills a specialized role in zebrafish that is not manifest in mammalian species, and that the two zebrafish PrP forms act in distinct cellular pathways or physiological processes. This may explain why fish express two PrP proteins, while higher vertebrates express only one. It is also worth noting that the peptide repeats in the N-terminus of zebrafish PrPs lack four conserved histidine residues that are responsible for copper binding in mammalian PrP [31], raising the possibility that fish PrP may lack some of the functional activities of PrP from higher species. Finally, it remains to be determined exactly how PrP-1 regulates E-cadherin trafficking. The authors argue against a direct, physical interaction between the two proteins, and instead suggest the involvement of signaling events triggered by PrP-1, for example tyrosine kinase activation induced by homophilic interactions between PrP-1 molecules on adjacent cells.

Can the results of the Málaga-Trillo et al. study be related to any previous observations on PrP functionality in mammalian systems? The cell adhesion function of zebrafish PrP-1 is reminiscent of previous observations made in mouse neuroblastoma cells and hippocampal neurons, supporting a role for mammalian PrP in cell–cell interactions and neurite outgrowth [32–34]. In addition, some neurodevelopmental phenotypes have been described in PrP knockout mice [16,35], which could be related to those observed in zebrafish embryos. Finally, there is evidence that mammalian PrP is capable of activating protein kinase-based, transmembrane signaling cascades that may be similar to those described in this study [21].

Aside from demonstrating the importance of PrP-1 and PrP-2 in morphogenesis of the zebrafish embryo, the new results may also have implications for understanding prion diseases. Of course, prion diseases are not developmental disorders, and their associated neuropathology is distinct from the PrP morphant phenotypes observed in zebrafish. However, if PrP^C^ plays a role in maintaining nerve cell contacts (synapses) in the adult brain, then loss of this function as a result of conversion to PrP^Sc^ could have deleterious effects that contribute to the disease state. Since some prion disorders are attributable to germline mutations in the PrP gene, it should be possible to test whether these pathogenic mutations cause a loss or gain of function phenotype in zebrafish. A fascinating question is whether zebrafish could be infected with prions. If so, then this organism could represent a powerful system for drug screening. Incidentally, fish for human consumption are sometimes fed with meat and bone meal [36], so the possibility of a “natural” prion infection in fish cannot be excluded. In conclusion, it seems likely that prion researchers will be hearing much more in the future from animals with fins as well as those with feet.

## Abbreviations

PrP: prion protein
PrP^C^: cellular form of PrP
PrP^Sc^: scrapie (infectious) form of PrP
S2: Schneider 2
