# Correction: Fishing for Prion Protein Function

**DOI:** 10.1371/journal.pbio.1001902

**Published:** 2014-06-13

**Authors:** The PLOS Biology Staff

The authors noticed an error in the Figure 2 legend. In the text, Ca^+2^ should read Ca^2+^. The authors have provided a corrected version here.

**Figure 2:**
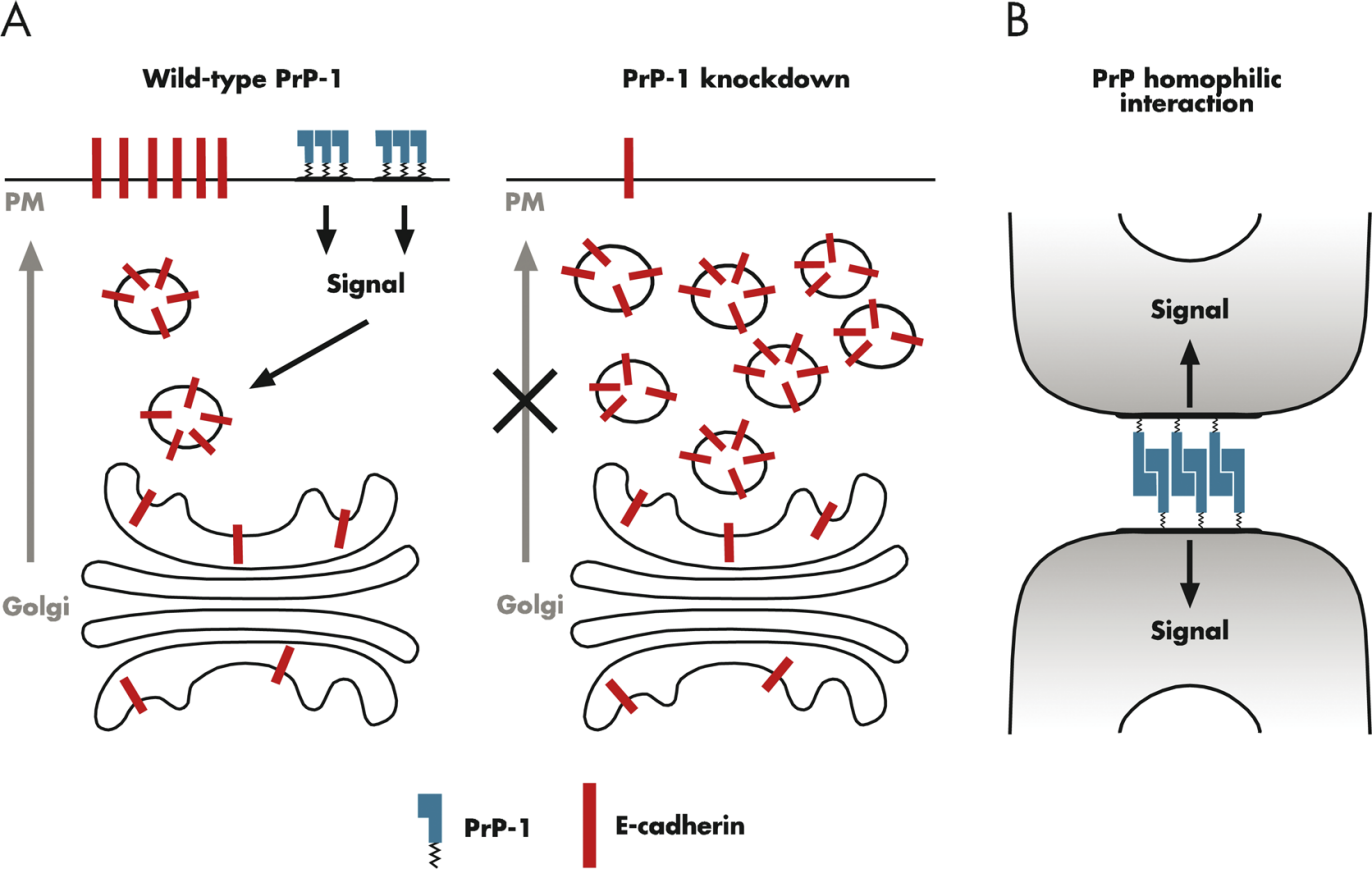
Two Roles for PrP in Cell Adhesion. (A) In wild-type zebrafish (left), PrP-1 promotes proper delivery of E-cadherin from the Golgi to the plasma membrane (PM), possibly via activation of a signal transduction cascade involving Src-family tyrosine kinases. In morphant fish lacking PrP-1 (right), E-cadherin accumulates in intracellular vesicles, resulting in reduced delivery to the plasma membrane. As a result, Ca^2+^-dependent, cadherin-mediated cell adhesion is impaired. (B) PrP molecules on adjacent cells undergo homophilic interactions that promote cell adhesion in a Ca^2+^-independent manner, at the same time generating an intracellular signal involving tyrosine phosphorylation. The PrP functions depicted in the two panels of this figure could be linked, if the intracellular signal generated by homophilic binding of PrP molecules (B) regulates cadherin trafficking (A).

## References

[1] Chiesa R, Harris DA (2009) Fishing for Prion Protein Function. PLoS Biol 7(3): e1000075 doi:10.1371/journal.pbio.1000075 10.1371/journal.pbio.1000075 PMC2661967 19338390

